# Mobile Robot + IoT: Project of Sustainable Technology for Sanitizing Broiler Poultry Litter

**DOI:** 10.3390/s24103049

**Published:** 2024-05-11

**Authors:** Alan Kunz Cechinel, Carlos Eduardo Soares, Sergio Genilson Pfleger, Leonardo Luiz Gambalonga Alves De Oliveira, Ederson Américo de Andrade, Claudia Damo Bertoli, Carlos Roberto De Rolt, Edson Roberto De Pieri, Patricia Della Méa Plentz, Juha Röning

**Affiliations:** 1 Graduate Program in Automation and System Engineering, Federal University of Santa Catarina, Florianópolis 88040-900, SC, Brazil; edson.pieri@ufsc.br; 2 Graduate Program in Food Sciences, Federal University of Santa Catarina, Florianópolis 88034-001, SC, Brazil; cesszootec@gmail.com; 3 Graduate Program in Computer Science, Federal University of Santa Catarina, Florianópolis 88040-900, SC, Brazil; 4 Undergraduate Program in Computer Science, Federal University of Santa Catarina, Florianópolis 88040-900, SC, Brazil; leoluizoliveira123@gmail.com; 5 Agronomy Department, Federal Institute of Paraná, União da Vitória 84603-264, PR, Brazil; ederson.andrade@ifpr.edu.br; 6 Graduate Program in Plant and Animal Science, Catarinense Federal Institute, Camboriú 88340-055, SC, Brazil; claudia.bertoli@ifc.edu.br; 7 Graduate Program in Business Management and Socioeconomic Science—ESAG, State University of Santa Catarina—UDESC, Florianópolis 88035-001, SC, Brazil; crderolt@gmail.com; 8 Biomimetics and Intelligent Systems Group, University of Oulu, P.O. Box 4500, 90014 Oulu, Finland; juha.roning@oulu.fi

**Keywords:** agro-industry 5.0, IoT, ozone, pesticide, poultry litter, robotic plataform, sensors

## Abstract

The traditional aviary decontamination process involves farmers applying pesticides to the aviary’s ground. These agricultural defenses are easily dispersed in the air, making the farmers susceptible to chronic diseases related to recurrent exposure. Industry 5.0 raises new pillars of research and innovation in transitioning to more sustainable, human-centric, and resilient companies. Based on these concepts, this paper presents a new aviary decontamination process that uses IoT and a robotic platform coupled with ozonizer (O_3_) and ultraviolet light (UVL). These clean technologies can successfully decontaminate poultry farms against pathogenic microorganisms, insects, and mites. Also, they can degrade toxic compounds used to control living organisms. This new decontamination process uses physicochemical information from the poultry litter through sensors installed in the environment, which allows accurate and safe disinfection. Different experimental tests were conducted to construct the system. First, tests related to measuring soil moisture, temperature, and pH were carried out, establishing the range of use and the confidence interval of the measurements. The robot’s navigation uses a back-and-forth motion that parallels the aviary’s longest side because it reduces the number of turns, reducing energy consumption. This task becomes more accessible because of the aviaries’ standardized geometry. Furthermore, the prototype was tested in a real aviary to confirm the innovation, safety, and effectiveness of the proposal. Tests have shown that the UV + ozone combination is sufficient to disinfect this environment.

## 1. Introduction

Society is experiencing a moment of transformation in both technology and ethical values. Care with people and environmental protection have a broader dimension nowadays. For this reason, clean technology solutions, those that ensure environmental preservation and people’s health, are well accepted in society and add value to the final product. These tendencies meet the concept of Industry 5.0 in which industries are concerned with environmental preservation and people’s wellness.

Industry 5.0 strictly relates to “Society 5.0”, a term developed in Japan in 2016. Society 5.0 attempts to balance economic development with solutions to combat societal and environmental problems by addressing social challenges built upon the union of physical and virtual domains. Society 5.0 is a society in which advanced IT technologies, Internet of Things (IoT), robots, artificial intelligence (AI), and augmented reality are actively present in everyday life, industry, healthcare, and other spheres of activity, not only for economic advantage but also for the benefit and convenience of each citizen [1].

Toxic compounds currently used in poultry farming to control endoparasites and ectoparasites are extremely bioaccumulative [2]. Because of that, they are responsible for inducing damage to the central auditory system [3] and might be involved in the etiology of Parkinson’s disease [4]. Alongside the toxic compounds’ collateral effect, records of these toxic ingredients appear in rivers, rainwater, subterranean water, animal and human milk, meat, honey, and vegetables consumed by humans [5,6,7,8].

An effective way to deal with this problem is to develop a solution that brings together clean technologies, automation, environmental safety, health, and well-being for producers through a new autonomous aviary decontamination process.

Nowadays, it is possible to use intelligent systems to monitor physical, chemical, and biological conditions in poultry farming environments. Using technologies such as ozone and ultraviolet lights makes it possible to efficiently control toxic residues in poultry litter, reducing the incidence of contaminants in commercial poultry raising [9]. Combining these technologies with a robotic platform presents a compelling approach for aviary decontamination.

This paper presents an open, low-cost, and sustainable technology for sanitizing broiler poultry litter, contributing to the sustainable production of the poultry sector. Also, it ensures the production standards required for food security through the design of an autonomous mobile robot coupled with ozone and UVL. The benefits mentioned above are in line with at least three UN Sustainable Development Goals/Targets:

- Target 2.4 [10]: “By 2030, ensure sustainable food production systems and implement resilient agricultural practices that increase productivity and production, that help maintain ecosystems, that strengthen capacity for adaptation to climate change, extreme weather, drought, flooding and other disasters and that progressively improve land and soil quality”.
- Target 2.a [10]: “Increase investment, including through enhanced international cooperation, in rural infrastructure, agricultural research and extension services, technology development and plant and livestock gene banks in order to enhance agricultural productive capacity in developing countries, in particular least developed countries”.
- Target 8.2 [11]: “Achieve higher levels of economic productivity through diversification, technological upgrading and innovation, including through a focus on high-value added and labour-intensive sectors”.
- Target 8.4 [11]: “Improve progressively, through 2030, global resource efficiency in consumption and production and endeavour to decouple economic growth from environmental degradation, in accordance with the 10-year framework of programmes on sustainable consumption and production, with developed countries taking the lead”.
- Goal 12 [12]: “Ensure sustainable consumption and production patterns”.

This research contributes twofold: 1. It defines a new process of decontamination and degradation of toxic compounds applied in poultry production. This new process relies on a unified solution composed of IoT devices, a dashboard system, and a mobile robotic platform that autonomously navigates inside aviaries while releasing an appropriate amount of ozone and UVL on the aviaries’ litter, ensuring proper decontamination. 2. It delivers the design of a custom mobile robot using open-source technologies and off-the-shelf components, thus ensuring access to poultry producers to this new technology.

We propose to utilize temperature, humidity, and pH sensors to ensure the effectiveness of the decontamination process. These sensors will provide crucial data for decision-making, particularly in detecting the optimal environment for the growth of pathogenic microorganisms. An Internet of Things (IoT) platform, comprising freely available open-source software, is employed to achieve this objective by continuously monitoring the poultry litter and issuing user alerts.

Users will operate the robotic platform through a mobile app responsible for starting and stopping the robot’s autonomous navigation. Also, the app will have an intuitive and friendly interface, simulating a joystick that will allow farmers and staff to control the robot manually. Moreover, it will serve as a robot status monitor, showing information about the ozonizer, UVL, battery, and task execution.

The robot’s control system will utilize the Robotic Operating System (ROS) to manage navigation processes, such as locomotion, localization, and sensor readings. We intend to develop a new ROS module to provide the mechanisms for controlling the amount of ozone and UVL released on the aviary floor.

The rest of this paper is organized as follows. Section 2 describes related works about ozone and UVL used for decontamination, robotic applications in the agricultural sector, and commercial robots regarding poultry farming. Section 3 describes the project of the new decontamination process of poultry litter, gives details about the architecture, and explains how farmers will perform their activities in the new process. Section 5 qualitatively compares the tradition and the new decontamination process. Finally, Section 6 concludes the paper and presents future research directions.

## 2. Related Works

Due to the multidisciplinary features of this project, this section is divided into biological and robotic subsections. The biological subsection covers research on poultry litter contamination, chicken diseases, and the efficacy of utilizing ozone and UVL for poultry litter decontamination. The second subsection shows areas where mobile robots are already widespread, the application of mobile robots in poultry farms, and challenges faced in mobile robot development.

### 2.1. Biological and Toxic Residue

The poultry farmer often works exposed to unhealthy environments, with risk factors associated with symptoms such as headache, eye or airway mucosal problems, difficulty breathing, and respiratory diseases [13]. The absence of personal protective equipment, mistaken dilution, and overdose can cause occupational and accidental poisoning in animals and humans.

Using pesticides from the Pyrethroid Chemical Group (neurotoxic pesticides) can cause intoxication and affect the peripheral auditory system regardless of exposure to noises [14,15]. The existence of houses or places to have meals near the accommodation for animals generates potential risk factors associated with oral and/or nasal contamination by *Staphylococcus aureus* [16]. Congenital malformations can be associated with exposure of the rural population to pesticides. Relevant and significant in public health problems, Cypermethrin is one of the active ingredients used in poultry farming and is also considered an endocrine disruptor [17,18]. As a result of monitoring women with a history of agricultural activities, it was observed that exposure to pyrethroids during pregnancy is associated with low birth weight [19]. Signs and symptoms of dizziness, headache, blurred vision, fatigue, cramps, weakness, paresthesias, and cognitive disorders (lack of concentration, mental disorders, and poor memory) were observed in rural workers when exposed to various types of pesticides from the pyrethroid group [20].

The degradation of toxic substances present in the poultry breeding environment depends on the intensity of solar radiation (as ultraviolet light and temperature), besides other environmental factors [21]. Hence, it is crucial to consider the physical, chemical, and biological processes of the soil biota and their environment. The degradation of pesticides often involves the decomposition of essential oils with repellent and insecticidal action [22,23]. Therefore, there is a need for safe and clean methods, such as ozone and UVL, for poultry environment decontamination, aiming to preserve the health of farmers and the environment.

Regarding pathogenic microorganisms, the bacteria *Escherichia coli*, one of the most sensitive microorganisms to exposure to ozone, reached 80% inactivation with low concentrations of ozone gas. Furthermore, it was described that ozone concentrations, pH, temperature, exposure time, degree of mixing, and organic compounds can also influence the survival efficiency of these microorganisms [24]. Other species such as *Salmonella typhimurium* are susceptible to O_3_. However, a humidity content of 30% or less is considered more effective in controlling pathogen levels in poultry litter. This shows that treatment with gaseous O_3_ is an effective strategy as a decontamination technique to ensure microbiological safety in poultry litter [25].

Several viral infections can be contracted inside the poultry house, often through vertical or horizontal transmission. There is evidence of several viruses in the poultry farming environment, including Newcastle Disease Virus, Infectious Anemia Virus, and Marek’s disease, that can interact with other pathogens [26,27]. The Newcastle disease virus, which affects poultry worldwide, is highly sensitive to O_3_ [28].

Treatment with O_3_ for the control of protozoa has been tested and shows satisfactory results in inhibiting the growth and infectivity of oocysts [29]. However, some species are highly resistant to UVL and ozone [30]. Further studies are needed to assess the action of ozone on protozoa that trigger diseases in industrial poultry. Hence, there is an urgent need for new, low-cost, and safe anti-Eimeria strategies [31].

Ozone is one of the most used decontamination methods in the food industry since it is fast and low-cost. The O_3_ half-life time varies from a few seconds to hours, and its stability depends on factors such as pH (aqueous O_3_) and temperature (O_3_ gaseous), where 10 °C results in a 43% half-life reduction. The half-life of O_3_ in atmospheric conditions is approximately 30 min and reduces at high temperatures and low pressures [32,33]. Ozone is safe and does not leave residue. It demonstrates its potential to control fungi and insects alongside degrading mycotoxins and pesticide residues. Regarding insects and mites, some studies reported the application of O_3_ to control these pests as an effective strategy that replaces other toxic compounds that leave residues [34].

Considering pesticide application on the poultry litter during several cycles of breeding (45 days), besides contaminating installations and litter, enables its presence as residues in poultry (meat), as well as the contact with staff responsible for application (poultry farmers). Its misuse in poultry farming can also leave residues in the facilities and their vicinity (soil, water, fauna, and flora) [35].

Industries are highly interested in using ozone gas for various purposes because it has high oxidant characteristics. When compared with other chemical compounds (e.g., hydrogen peroxide, permanganate, and hypochlorite), ozone inactivation rates are faster against pathogenic microorganisms, react with many classes of compounds, and have a low production cost [36,37]. Since ozone is considered an effective sterilant for several microorganisms, many studies describe it as a safe alternative to inactivate several pathogens, such as bacteria, fungi, and viruses [38,39,40].

In addition to ozone gas, ultraviolet radiation from UV lamps stands out as another possible solution. When applied correctly, UV radiation does not generate waste in the environment and effectively reduces the microbial count. This ability to inactivate microorganisms has been demonstrated in several research works related to the germicidal effects of UV radiation [41,42]. Using UV radiation as a decontaminating agent is mainly attributed to UV-C rays, with wavelengths of 200–280 nm and a range of 250 to 260 nm. In this setting, UV-C rays are lethal to most microorganisms, including bacteria, viruses, protozoa, fungi, and algae [43].

### 2.2. Robotics

Mobile robots have been a popular research area in the last few years. The main reason is that mobile robots can perform tasks while autonomously navigating within the environment. Therefore, they can replace humans in repetitive or dangerous tasks that require mobility.

Due to mobile robots features, their applications may include [44] surveillance, planetary exploration, patrolling, emergency rescue operations, reconnaissance, petrochemical applications, industrial automation, construction, entertainment, museum guides, personal services, intervention in extreme environments, transportation, and medical care, and the list grows daily.

Mobile robots have already been a successful use case in warehousing scenarios, as the Kiva robot [45] due to its reduced costs and capability to increase performance in order preparation. The Kiva robots compound a multi-robot system in that each robot carries shelves, thus bringing inventory items to warehouse workers, which makes orders fulfilled faster. This system uses a distributed-control approach that enhances robots’ navigation capabilities, which reduces their mechanical complexity and price. This example reinforces that mobile robots performing transportation tasks within industrial and warehousing environments can increase efficiency while substantially reducing operational costs [46].

Robotic systems applications in agricultural field operation include weeding, seeding, spraying, and harvesting [47]. However, mobile robots are under-researched in poultry applications, especially those related to broiler chicken breeding. Works focused on poultry farms have been mainly intended for egg collection robotic systems, as in [48,49,50]. Among the challenges this type of application faces are autonomous navigation, obstacle avoidance, egg detection using image recognition, and sorting eggs without damaging them. On the other hand, in poultry houses for broiler chickens, mobile robotic systems must deal with sanitation and aeration of litter, promote animal welfare, detect conditions that can prejudice production, emit alerts about dead chickens, and so on.

In the market for poultry farming, there were two mobile robots and one ceiling-suspended robot available. The first mobile robot is the XO from Octopus Biosafety [51]. Its manufacturers reported that the robot helps poultry farmers to prevent risks and treat contamination. It performs scarification of the litter and cleaning by spraying sanitizing solutions. It monitors animals and the environment by measuring temperature, humidity, and ammonia. It also detects, counts, and localizes dead chickens. This solution is proprietary, and the research community does not have access to the code or the robot design.

The second mobile robot is the Sputinic NAV developed by Tibot [52]. Its developers claim it can perform indoor navigation along a self-generated or chosen route. Also, it stimulates the movement of poultry while aerating the litter using a scraper without turning it over completely. Since it does not apply any sanitizer in the litter, just scarifying it could bring pathogens to the surface.

The ceiling-suspended robot is the ChickenBoy from Faromatics [53]. According to its manufacturer, this solution autonomously monitors broiler welfare, making measurements of thermal comfort, air quality, health and welfare of animals, and proper farm equipment function. Farmers, vets, and stock persons interact with the robot through mobile alerts or a cloud platform. Once again, this is one more proprietary solution closed to the academic community.

The existing literature shows that proposals and developments are absent for open-source, low-cost mobile robots designed for disinfection processes in aviaries. Currently, the academic community lacks a foundational project to support researchers in their analysis and improvement efforts in this subject. This paper addresses this gap by presenting a new decontamination process utilizing IoT devices and a robotic platform developed with open-source technologies and readily available components. This project aims to provide the academic community with a framework for discussing potential advancements in this research domain. The following section outlines our proposal in detail.

## 3. New Decontamination Protocol Proposal for Poultry Using IoT and a Robotic Platform with an Ozonizer and UV Lights

In the face of the challenges looking for the best way to decontaminate aviaries using clean technologies, we propose a new decontamination protocol for poultry using IoT and a robotic platform with an ozonizer and UV lights. Because this project is a multidisciplinary research, we organized it into four stages: 1. Design and development of poultry litter sensors; 2. Design and development of a prototype robotic platform coupled with ozone and UVL; 3. Coding of the navigation algorithm with aviary disinfection; 4. Definition of a new decontamination protocol for poultry using the proposed platform. Each of these steps is detailed below.

### 3.1. Design and Development of Poultry Litter Sensors

One challenge during broiler breeding is to ensure healthy poultry litter. Several problems, as cited in related work, originate from contamination in the poultry litter. The development of pathogenic microorganisms such as bacteria, fungi, and viruses is related to temperature, pH, humidity, and ammonia levels. For example, fungi were identified using the following rules

- *Aspergillus flavus*: the development of this fungus occurs when the temperature of the poultry litter is between 25 and 29 °C and 30 to 85% humidity [54];
- *Echerichia coli*: when poultry litter pH decreases from an average of 8.0 to 3.0, the bacterial load also decreases [55];
- *Salmolella* sp.: they grow at an optimal temperature of 37 °C, but there have already been growths in temperatures between 5 and 45 °C, still growing at pHs ranging between 4 and 9, with optimal growth at pH 7 [56];
- *Clostridium perfringens*: there is a reduction at a pH range from 8.2 to 8.6, which hinders the germination of spores [55].

**Figure 1:**
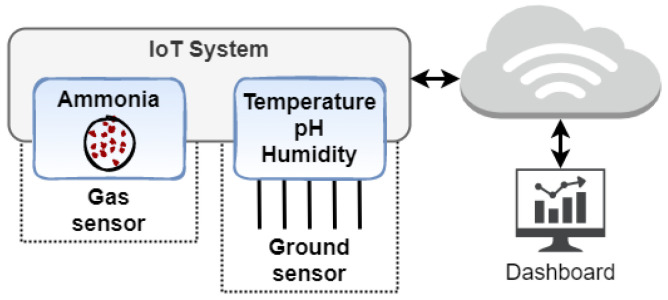
Poultry litter IoT platform.

Figure 1 illustrates the IoT platform developed for real-time evaluation of poultry litter. This platform comprises gas and soil sensors, enabling the prediction of poultry diseases through measurements of temperature, pH, humidity, and ammonia levels. In addition to sensor design, a dashboard is crucial for recommendations to technical managers regarding poultry litter management.

It is expected to use an MQ-137 analogic gas sensor and a soil sensor in the IoT platform prototype. According to its datasheet in [57], this low-cost sensor can detect concentration scope from 5 to 500 ppm of NH_3_. Its precision and resolution depend on the load circuit and the analog converter used to read sensing data, which will be determined in the future. However, although this kind of sensor has ammonia concentration measurements compared to a high-accuracy instrument, it requires constant calibration according to relative humidity [58]. Hence, this matter necessitates consideration during IoT platform development. The soil sensor is fixed on the ground to measure humidity, temperature, and pH. According to its datasheet, it uses five stainless steel probes to measure soil moisture (humidity), temperature, and pH. Table 1 summarizes parameters from this sensor obtained in the datasheet [59].

Technical managers will have real-time information about the litter using data generated by the sensors. Therefore, they can make accurate decisions and guide poultry farmers to use the decontamination mobile robot to prevent diseases or improve poultry wellness in the aviary site. Using multiple sensors allows the poultry litter decontamination aiming at areas where the conditions are critical.

### 3.2. Design and Development of the Prototype Robotic Platform Coupled with Ozone and UVL

**Figure 2:**
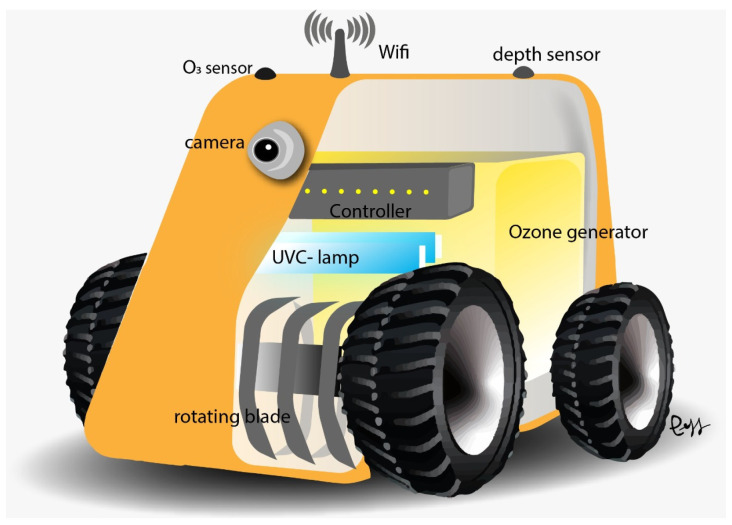
Artistic representation of the robotic platform’s front view.

The design of the robotic platform prototype considers the use of low-cost materials and open technologies. Figure 2 is an artistic representation of the front view of the robotic platform, consisting of wheels, rotating blades, sensors (camera, depth sensor or LIDAR, and O_3_ sensor), and the ozone and UVL devices.

**Figure 3:**
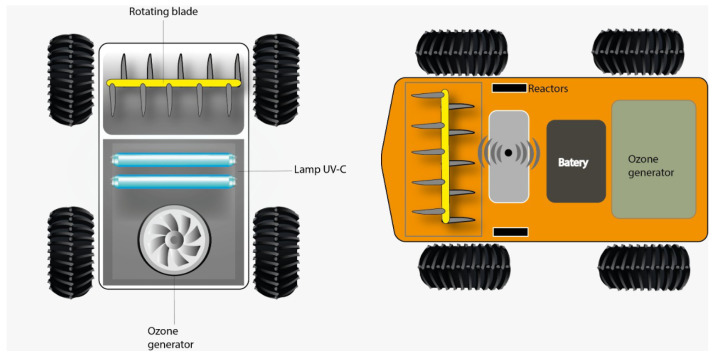
Robotic platform overview.

Figure 3 shows the robotic platform overview. The left side depicts the poultry litter turner (rotating blade), the UVL bulbs, and the ozonizer device. The right side illustrates the robotic platform’s top view with battery, WiFi device, and other hardware components.

Poultry farmers in developing countries lack funding support to acquire high-cost robotic platforms. Consequently, this project prioritizes the development of a low-cost robotic platform. This goal is attainable by utilizing off-the-shelf components, including the Raspberry Pi 4, webcam, and DC motors. However, the feasibility of incorporating a LIDAR sensor, the most expensive component of the mobile robot, is still under investigation.

**Figure 4:**
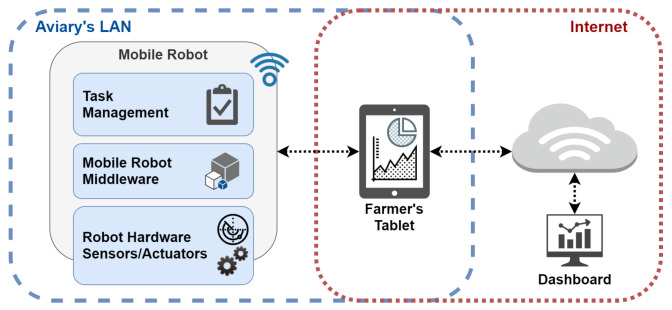
Robotic platform’s communication architecture.

Figure 4 illustrates the communication architecture of the robotic platform proposed in this study. It is assumed that the poultry house is equipped with a Local Area Network (LAN) accessible via a Wi-Fi router. Consequently, the mobile robot connects to this LAN, enabling the farmer to control it via a tablet. Even when the router lacks internet connectivity, the robot can receive commands from the farmer’s tablet over the LAN.

The robot’s task management system receives commands from a farmer’s tablet via Wi-Fi. Subsequently, utilizing a mobile robot middleware, the robot will be capable of environmental sensing, reading proprioceptive data (e.g., battery charge and sensor status), and controlling actuators to execute aviary sanitization.

Data produced by the robot are sent to the farmers’ tablet and then forwarded to the database, which populates the dashboard. These data encompass the decontamination status (completed or failed) and logs of the robot’s sensors. Despite the autonomous capabilities, a human must oversee the decontamination process for safety reasons. Therefore, the tablet-stored data undergo validation by the user and then are forwarded to the database at the end of the disinfection. Although being a well-known software architecture, it creates some challenges regarding filtering and storing robot data. The mobile robot middleware poses new questions to the academic community because, beyond proprioceptive data, robots also have navigation data, fault tolerance aspects, security, and real-time constraints. All these aspects are addressed within this software architecture.

We use the ROS (Robotic Operating System) as the mobile robot middleware. It is an open-source meta-operating system that provides a core of components that facilitate the development of solutions for mobile robots. The main ROS components are message passing, recording, and playback of messages, remote procedure calls, and distributed parameter systems. ROS also provides a set of well-known algorithms used in robotics for navigation. All these components are used in the proposed solution, as described in Section 3.3.

### 3.3. Navigation Algorithm for Aviary Disinfection

**Figure 5:**
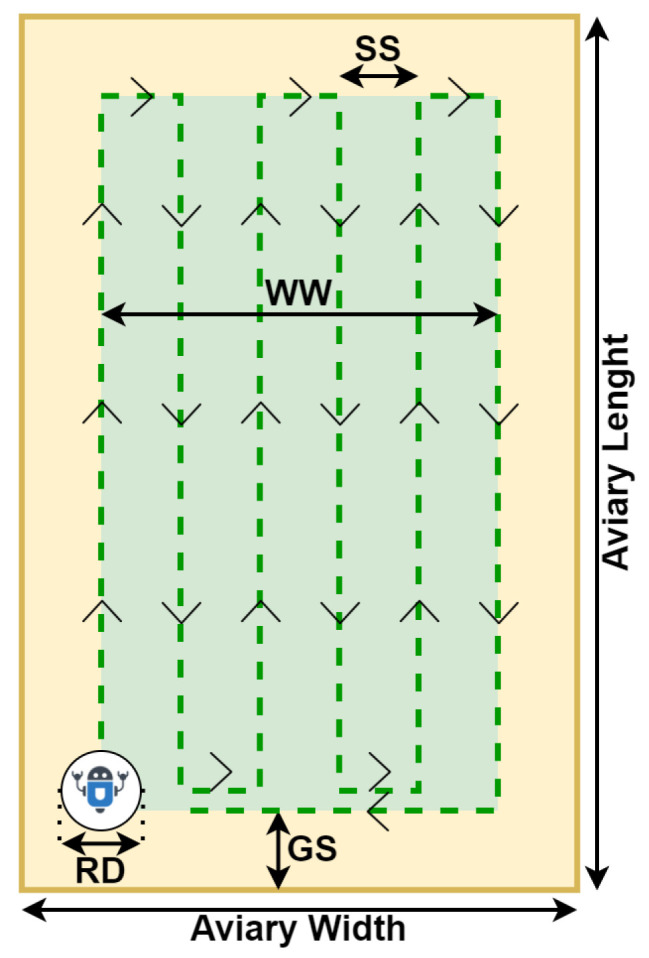
Representation of the robot’s path on the aviary. Yellow is the total area of the aviary. Green is the area covered by the robot. SS stands for step size. WW means working width. RD represents the robot’s diameter and GS stands for the guardrail size.

The robot will use maneuvers similar to the ones used to cut grass using a lawnmower to turn over the poultry litter and apply the gas ozone. As shown in Figure 5, the system can consider a working area smaller than the total area of the aviary. Some aviaries can have cement sidewalks on the edges. Therefore, if the system does not consider a working area, the robot could try to turn over the sidewalk instead of shavings. Also, the path always starts in one corner of the working area, starting and ending at the same point in the environment. Figure 5 depicts an aviary featuring standardized geometry, which serves as the framework for designing the robot’s path planning.

As seen in Figure 5, the navigation path is described in terms of the step size parameter. The user computes this parameter using guardrail size, the minimum and maximum percentage of accepted overlap in the path, and the robot’s scarifier diameter. Then, the system defines the working width using Equation (1). After that, it calculates both the minimum and maximum number of steps used to define the step size (Equations (2) and (3)). Equation (6) defines the minimum number of steps. The last line of the path will have the sense directed to the starting position. Finally, the step size is given by Equation (7).

(1) $$\begin{array}{c} WW=AW-2\times GS \end{array}$$

(2) $$\begin{array}{c} MiNS=\left\lfloor \frac{WW}{(1-MiPO)\times RD}\right\rfloor \end{array}$$

(3) $$\begin{array}{c} MaNS=\left\lceil \frac{WW}{(1-MaPO)\times RD}\right\rfloor \end{array}$$

(4) $$\begin{array}{c} MaPO,MiPO\in (0,1) \end{array}$$

(5) $$\begin{array}{c} MaPO-MiPO\geq 0.15 \end{array}$$

(6) $$\begin{array}{c} NS=MON(MiNS,MaNS) \end{array}$$

(7) $$\begin{array}{c} SS=\frac{WW}{NS} \end{array}$$

where

- WW: work width;
- AW: aviary width;
- GS: guardrail size;
- MiNS: minimum number of steps;
- MaNS: maximum number of steps;
- RD: robot’s scarifier diameter;
- MiPO: minimum path overlap;
- MaPO: maximum path overlap;
- NS: number of steps;
- MON: minimum odd number between two numbers;
- SS: step size.

The first line of the path must be parallel to the aviary length. Depending on the robot step size, the coverage will purposefully overlap a portion of the already covered path. The overlaps are already manually carried out by workers to not create regions similar to speed bumps in the poultry litter. Furthermore, the ozone gas will spread on the surface of the poultry litter. Overlapping the path is a way to re-apply ozone in the edges of the area covered by the ozonizer.

#### Preliminary Experiment

A simulation was conducted using Gazebo to validate the suitability of ROS as middleware software for this architecture. Gazebo is an open-source multi-robot simulator introduced in [60]. Further details about the simulator can be accessed at http://gazebosim.org/ (accessed on 5 November 2022).

**Figure 6:**
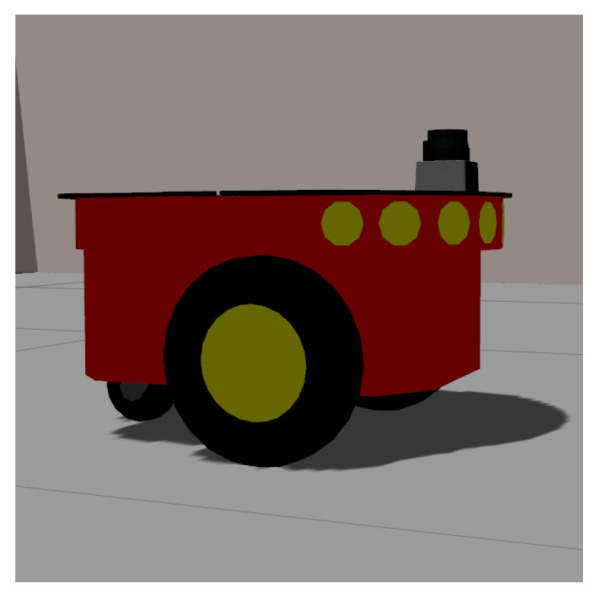
Pioneer 3-DX mobile robot used in the simulation.

Figure 6 shows the robotic platform used in the simulation, a Pioneer 3-DX (manufactured by Adept MobileRobots, Amherst, EUA). It has a 455 mm length, 381 mm width, 237 mm height, and wheels of 195 mm diameter. The robot was equipped with a LIDAR sensor with a 5 m range and 180-degree aperture in the simulation.

**Figure 7:**
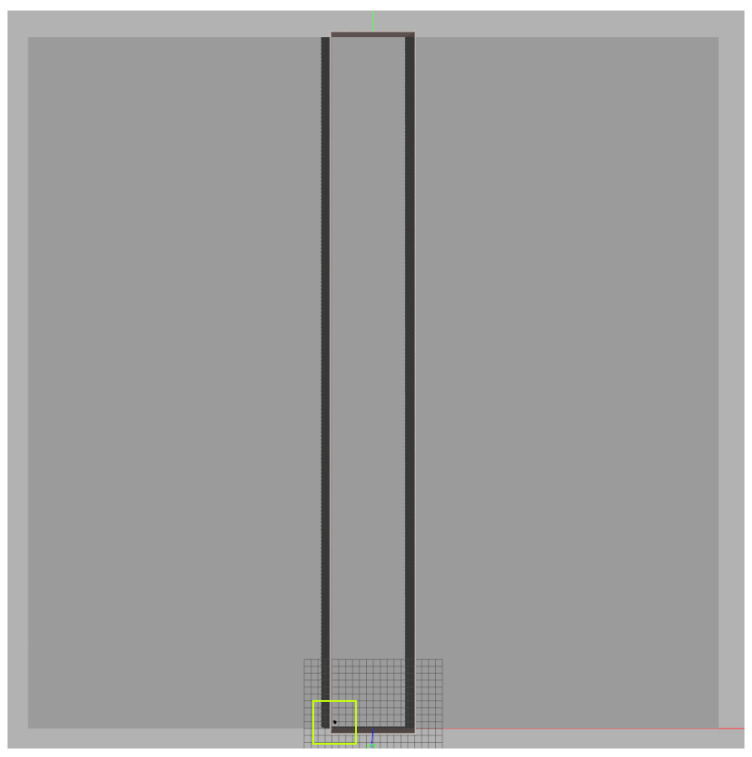
Top view of the simulation. The small dot inside the green square is the robot. The environment is much larger compared to the robot.

Figure 7 represents a typical aviary with a standard size within the simulation. It has 100 m length, 12 m width, and 1 m guardrail. The generated path considered a robot with a scarifier diameter of 1.1 m, minimum path overlap of 0%, and maximum path overlap of 15%. The following path Equations were used:

(8) $$\begin{array}{c} WW=AW-2\times GS=12-2=10 \end{array}$$

(9) $$\begin{array}{c} MiNS=\left\lfloor \frac{WW}{(1-MiPO)\times RD}\right\rfloor =\left\lfloor \frac{10}{(1-0.0)\times 1.1}\right\rfloor =9 \end{array}$$

(10) $$\begin{array}{c} MaNS=\left\lceil \frac{WW}{(1-MaPO)\times RD}\right\rceil =\left\lceil \frac{10}{(1-0.15)\times 1.1}\right\rceil =11 \end{array}$$

(11) $$\begin{array}{c} NS=MON(MiNS,MaNS)=MON(9,11)=9 \end{array}$$

(12) $$\begin{array}{c} SS=\frac{WW}{NS}=\frac{10}{9}\approx 1.11 \end{array}$$

**Figure 8:**
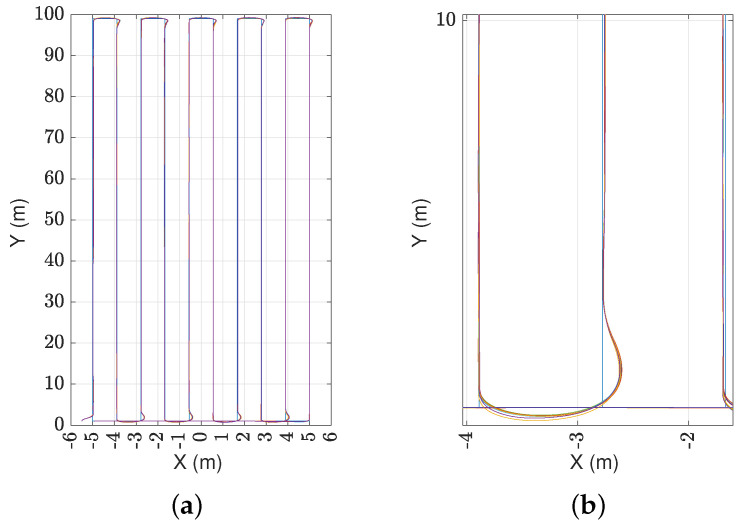
Result from the path execution experiment. (**a**) Global path. (**b**) Zoom in from Figure (**a**). Blue is the expected path.

Different from the poultry sheds, the simulation has no terrain irregularities. The experiment was repeated ten times, and the expected and obtained paths in each experiment’s execution are shown in Figure 8. The paths are very close to each other, which shows stability in the system. The biggest errors occur on end-of-line curves, reaching 18 cm and 4 cm for *X* and *Y* coordinates, respectively. However, in a physical and irregular terrain, there probably will be bigger variations between executions. One of the advantages of ROS is its simplicity in redefining parameters for real scenarios. As the ROS is organized into core components, it is easy to use specific components in the proposed software architecture.

### 3.4. Definition of a New Decontamination Protocol for Poultry Using the Proposed Platform

**Figure 9:**
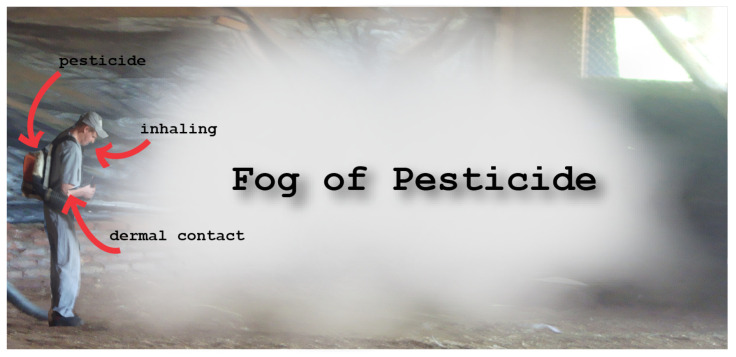
Traditional decontamination model.

Figure 9 depicts the traditional decontamination process, where producers monitor the aviary’s conditions daily. If they detect strong odors, excessive humidity, or the presence of insects, they must turn over the poultry litter. This task can be performed while the poultry remains inside the aviary. Eventually, it is also necessary to spread pesticides throughout the aviary. In this case, the poultry must be removed from inside the aviary. After that, the worker must mix the proper pesticide dosage, place it in a compartment (coastal atomizer), and spread it. The safety equipment is often not used. As the poultry farmer has other agricultural activities for their subsistence, they do not use safety clothing because of the hurry to perform the other activities. Another reason is the costs of the equipment that burden the poultry farmer.

**Figure 10:**
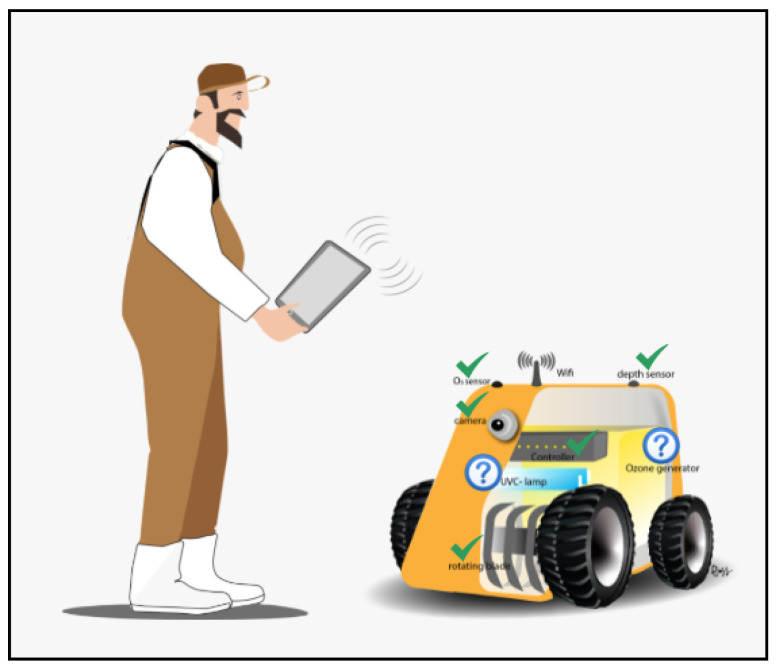
Robotic platform checking.

All information about the poultry litter will be accessible on the tablet under the new decontamination process. With these data readily available, the farmer can determine whether immediate decontamination is necessary. If so, the farmer will also use the tablet to check that all functionalities of the robotic platform are operational, such as ozone levels and battery status, as shown in Figure 10. Once these preparations are complete, the farmer will remove the poultry from the aviary and deploy the mobile robot.

**Figure 11:**
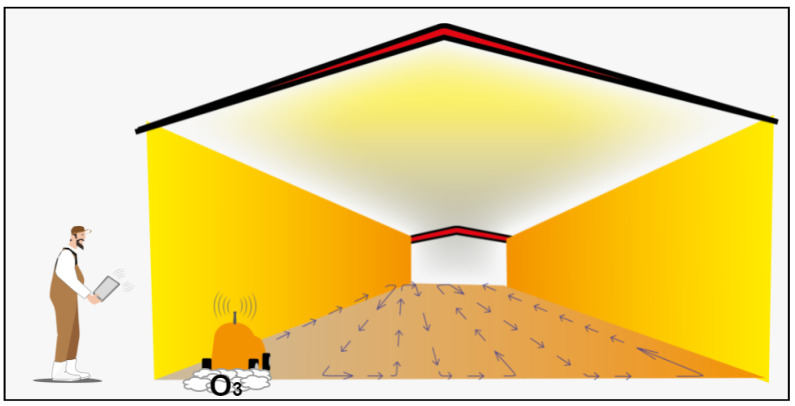
Poultry litter decontamination.

Figure 11 illustrates that during the decontamination process, the farmer is not allowed to enter the aviary, as the robot will be emitting ozone and UV light. The farmer can monitor the robot’s movements from outside the aviary using a camera mounted on the robotic platform. Currently, studies are underway to determine the optimal concentrations and exposure durations of ozone gas for various conditions of poultry litter. Ongoing research and testing aim to establish precise and efficient methods for applying ozone gas in poultry units.

**Figure 12:**
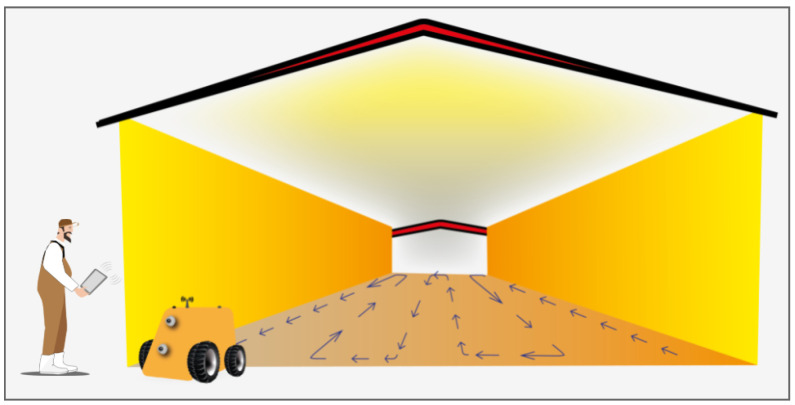
Decontamination conclusion.

Figure 12 presents the robotic platform returning to the entrance to the aviary at the end of the decontamination process. The farmer must switch it off, clean it, and then store the robot for the next use.

The machinery used on one poultry litter must be cleaned before it is used on another to prevent cross-contamination in the traditional protocol. Similarly, the robot must be cleaned after use following the same rationale. However, the robot offers an advantage: it self-decontaminates as it is continuously exposed to ozone throughout its operation. Despite this benefit, ozone exposure increases the robot’s susceptibility to metal oxidation. The robot’s design will include hermetically sealed circuits and metal components coated with protective paint to reduce the oxidation vulnerability. Nonetheless, regular preventive maintenance is essential to keep the robot in optimal working condition.

## 4. Robotic Platform Prototype

**Figure 13:**
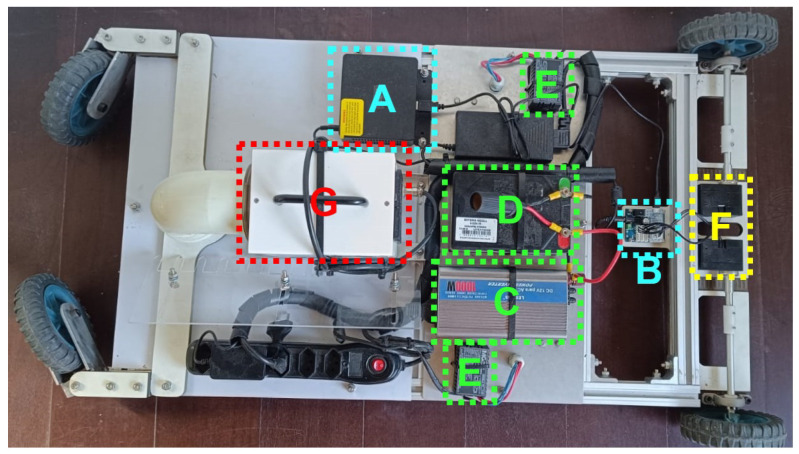
Robotic platform prototype—top view.

In this section, we present the robotic platform prototype built to validate the combination of ozone and UVL as a sanitizer agent in the poultry house scenario. Figure 13 shows the top view from the robotic platform prototype. The elements related to control are indicated in cyan, in green are power supply components, in yellow are the actuators, and in red are the sanitizers.

**Figure 14:**
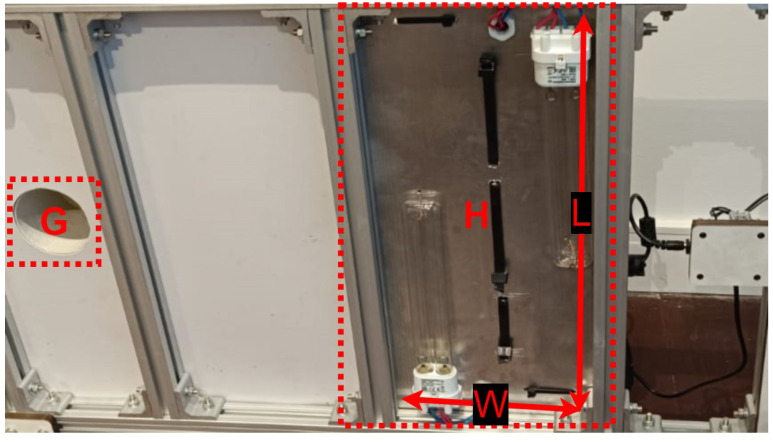
Robotic platform prototype—bottom view.

**Figure 15:**
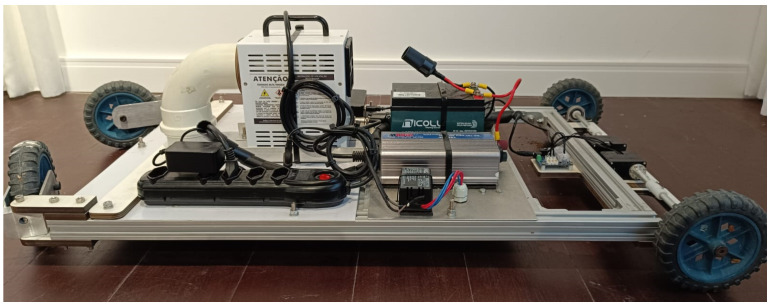
Robotic platform prototype—side view.

Figure 14 shows the bottom view from the robotic platform prototype. It follows the same color scheme previously mentioned. Additionally, Figure 15 shows the side view from the robotic platform.

Following, each one of the components is described with their specifications:

- Control:
  **A →** Mini PC Beelink with processor Intel Celeron J4125 and 8 GB RAM
  **B →** U2D2 + U2D2 Power Hub Board: modules used for power supply and communication with the motors
- Power Supply:
  **C →** Voltage inverter: 12 V CC to 220 V AC
  **D →** Battery: 12 V @ 20 Ah
  **E →** Electronic reactor 20 W: used by the UV-C
- Actuators:
  **F →** Two motors DYNAMIXEL XM540-W150-T
- Sanitizers:
  **G →** WIER OZmini Ozone generator: produces 1 g/h of ozone from air
  **H →** UV-C lamps OSRAM Puritec 18 W

We selected a UV-C lamp with enough power to kill *Salmonella* spp. and *E. coli*, two common microorganisms in poultry litter. The UV-C dose required for killing Salmonella is around 152 J/m^2^, and *E. coli* is about 66 J/m^2^ [61]. In order to select the proper lamps, we used the following equations.

The UV-C dose (Equation (13)) is given by the irradiance (*I*) and the time of exposure (*t*). The irradiance (Equation (14)) is given by the power of the lamp (*P*) and the lamp’s area of reflection (*A*), which can be represented by L·W considering the area in Figure 15 marked with **H**.

(13) $$D=I\cdot t\to t=\frac{D}{I}$$

(14) $$I=\frac{P}{A}=\frac{P}{L\cdot W}$$

Considering that the robot travels at a constant average velocity for the robot and Equations (13) and (14), we can estimate the minimum power for the UV-C lamp. Using Equation (15) and considering that the robot moves on average at 0.25 m/s, we need a UV-C lamp with at least 15.2 W.

(15) $$velocity=\frac{distance}{time}=\frac{W\cdot I}{D}=\frac{W\cdot P}{D\cdot L\cdot W}=\frac{P}{D\cdot L}\to P=velocity\cdot D\cdot L$$

(16) P=0.25×152×0.4=15.2W

## 5. Evaluation of the New Decontamination Protocol Proposal and the Initial Results

Industry 5.0 redefines how humans and robots work together, adding more value to automation processes. It arises from the consolidation of 5G networks, the evolution of cloud computing (especially fog computing), and technological advancement. The advances achieved by sensors play a fundamental role in the maturation of this new industry paradigm.

The proposal presented in this article is based on the concepts of Industry 5.0. It seeks to place people at the center of processes, improving the following aspects of life: inclusion, sustainability, and quality of life. In this sense, the proposed decontamination process is innovative because it preserves the physical integrity of the poultry farmer, avoiding exposure to toxic compounds currently used. Therefore, the proposed decontamination process is sustainable and provides quality of life for poultry farmers. It is also inclusive because it puts the farmer in charge of the house’s decontamination process.

The Internet of Things (IoT) plays a key role in this application. Information captured by sensors installed in the poultry litter will make the robot’s action more accurate and effective in decontaminating the poultry litter. The operator will be able to anticipate the decontamination management before litter conditions become harmful to the broilers and the farmer. In this project, we are using humidity, pH, and temperature sensors. The data generated by the sensors deliver real-time information to the poultry farmer so that he knows the real conditions of the aviary.

The next subsection presents the initial results achieved by our research.

### Assessment of the Initial Results

We evaluated the prototype within an aviary, sampling and assessing the poultry litter for each scenario. We partitioned a section of the aviary into cells, and the robot navigated through each of these cells in three distinct manners:

1. First group of cells in which the robot only had the ozone on (UV light off);
2. Second group of cells in which the robot had only the UV light on (ozone off);
3. Third group of cells in which the robot had ozone and UV light turned on at the same time.

After the disinfection process, the poultry litter was carefully collected from each group of cells and promptly transported to the laboratory for microbiological analysis. The microorganisms were evaluated in colony-forming units per milliliter of solution (CFU/mL). Table 2 shows the first column with the analyzed microorganisms, the second column with the methodology applied, and the third column presents the control values (poultry litter without disinfection process). The last three columns show different disinfection processes (UV light, ozone, and UV light + ozone).

It is crucial to note that UV light alone is not sufficient for disinfecting poultry litter. The disparity between disinfection by UV light and the control group is minimal. Similarly, relying solely on ozone application falls short of achieving effective disinfection. However, when UV light is combined with ozone, as indicated in the last column, a noticeable improvement in disinfection levels is observed. Overall, the UV light and ozone combination yielded the most favorable results.

The results presented in this research bring innovation along an effective and safe disinfection process. Furthermore, as it uses clean technologies such as ozone and ultraviolet light in the disinfection process, it preserves the environment and poultry farmers’ health. All of these factors improve the quality of life of poultry farmers.

We highlight the results of this research:

1. Definition of a low-cost robotic platform specific for aviary decontamination composed of the wheels, rotating blades, controllers, batteries, sensors, UV lights, and the ozone application device;
2. Experimental tests were carried out to measure soil moisture, temperature, and pH, establishing the range of use and the confidence interval of the measurements;
3. Create a software architecture to accommodate the IoT devices (sensors and tablets), mobile robot, and a dashboard;
4. Give specific features to the robotic platform’s communication architecture composed of a LAN which connects the mobile robots with the farmer’s tablet and dashboard;
5. Validation of the ROS navigation packages to perform decontamination path considering the longitudinal movement within the aviary, allowing reducing the number of turns. With this decision, energy consumption is reduced, and the robot’s navigation becomes simple;
6. Definition of the step size parameter which describes the navigation path;
7. Creation of a new decontamination protocol under Industry 5.0, contributing to the sustainable production of the poultry sector;
8. Simulation with Gazebo to validate all previous project decisions made to analyze the main characteristics for the unified approach considering both the robotic device and the proposed disinfection protocol.
9. Carrying out tests in a physical environment with a functional prototype where it was possible to prove the innovative, effective, and safe disinfection process.

## 6. Conclusions

More than ever, society has been pursuing ecologically correct technologies to reduce and mitigate food and environmental contamination, preserving natural resources and the health of rural producers.

This research touches on an important UN Sustainable Development Goal: Ensure sustainable consumption and production patterns. Once the world population is continuously growing, reaching other UN goals, including no poverty and zero hunger, requires ensuring that food production becomes cheaper, efficient, and sustainable. In the same way, as defined by the EU Commission, Industry 5.0 recognizes the power of industry to achieve societal goals beyond jobs and growth. It aims to become a resilient provider of prosperity by making production respect the boundaries of our planet. Additionally, it places the well-being of the industry worker at the center of the production process [1].

This work proposed an approach using IoT and mobile robot technologies to monitor and sanitize poultry litter. Our solution employs clean technologies, different from the traditional approach that uses pesticides. The IoT solution provides real-time information about poultry litter, allowing farmers and technicians to follow the evolution of litter conditions. Using a dashboard, they can receive recommendations related to preventive actions, which can maximize production and promote animal welfare. Based on the dashboard information, the mobile robot scarifies and decontaminates the poultry litter using ozone and UVL. When producers adopt this technology on a large scale within an integrated cloud system, it will enable the generation of big data. Employing data analysis processes will help establish the best breeding practices while correlating with climate data and workers’ health. Furthermore, it facilitates the application of prediction and machine learning techniques, among others, thereby shaping the upcoming challenges of the project.

Preliminary results showed that ROS is an adequate middleware to support the development of this solution. We validate ROS’s navigation packages to perform the decontamination path. The aviaries’ geometry increases the potential for reducing energy consumption. Therefore, we defined a set of equations for the path’s waypoints, resulting in fewer turns and less energy consumption.

In summary, the achievements are as follows: 1. The definition of an open low-cost robotic platform that uses proven technology to eliminate pathogenic microorganisms in aviaries; 2. Avoiding the use of non-friendly environmental substances like pesticides in the disinfection process inside aviaries; 3. Protection of farmers’ lives against diseases; 4. Proposition of an automatized solution combining robotics, IoT resources, ozone, and UV devices; 5. Simulation tests that show the viability of the proposed solution; 6. Tests with the functional prototype validated the effectiveness of the disinfection process against different microorganisms, which showed that combining UV and ozone is more effective in disinfecting poultry houses than their isolated usage.

The proposed solution could significantly reduce poultry farmers’ exposure to harmful gases and toxic compounds within the aviary environment, mitigating the risk of diseases such as cancer. Additionally, this solution could reduce the risk of respiratory and skin ailments caused by dust, mites, and microorganisms, thus promoting better health and well-being among farmers.

## Figures and Tables

**Table 1:** Parameters from the soil sensor.

Sensor	Range	Resolution	Precision
Humidity	0∼100%	0.1%	2% for 0∼50%
			3% for 50∼100%
Temperature	−40∼80 °C	0.1 °C	±0.5 (at 25 °C)
pH	3∼9	0.1	±0.3

**Table 2:** Microorganisms evaluated, methodology and results.

Microorganisms Evaluated	Methodology	Control (CFU/mL)	UV (CFU/mL)	Ozone (CFU/mL)	UV + Ozone (CFU/mL)
Gram-negative	Agar MacConkey	$28.0\times 10^{4}$	$24.3\times 10^{4}$	$12.2\times 10^{4}$	$9.1\times 10^{4}$
Total aerobes	Nutrient Agar	$18.5\times 10^{5}$	$16.0\times 10^{5}$	$16.0\times 10^{5}$	$8.8\times 10^{5}$
Enterobacteria	XLD Agar	$17.1\times 10^{5}$	$9.9\times 10^{5}$	$8.5\times 10^{5}$	$8.9\times 10^{5}$
*E. coli*	-	Present	Present	Present	Present
Salmonella	-	Missing	Missing	Missing	Missing

## Data Availability

No new data were created or analyzed in this study. Data sharing is not applicable to this article.
